# Blood feeding behaviour comparison and contribution of *Anopheles coluzzii* and *Anopheles gambiae,* two sibling species living in sympatry, to malaria transmission in Alibori and Donga region, northern Benin, West Africa

**DOI:** 10.1186/s12936-018-2452-9

**Published:** 2018-08-22

**Authors:** Martin C. Akogbéto, Albert Sourou Salako, Fortuné Dagnon, Rock Aïkpon, Michelle Kouletio, Arthur Sovi, Michel Sezonlin

**Affiliations:** 1 Centre de Recherche entomologique de Cotonou (CREC), Cotonou, Benin; 2 Faculté des Sciences et Techniques de l’Université d’Abomey-Calavi, Abomey-Calavi, Benin; 3 US President’s Malaria Initiative, US Agency for International Development, Cotonou, Benin; 4 Université Nationale des Sciences, Technologies, Ingénierie et Mathématiques, Abomey, Benin; 5 PMI VectorLink Project, Abt associates, Bamako, Mali

**Keywords:** *Anopheles coluzzii*, *Anopheles gambiae*, Sporozoite index, Entomological Inoculation Rate, Alibori, Donga, Benin

## Abstract

**Background:**

The main goal of this study was to assess the blood feeding behaviour and the contribution *Anopheles coluzzii* and *Anopheles gambiae*, 2 sibling species of *An. gambiae* sensu stricto. present and living in sympatry in 2 regions of northern Benin targeted for indoor residual spraying (IRS).

**Methods:**

The study was carried out in 6 districts of 2 regions of Benin (Alibori and Donga). Human landing catches (HLC) performed inside and outside of the households and pyrethrum spray captures (PSC) carried out in bedrooms were used to sample vector populations (*An. gambiae* and *An. coluzzii*). Collected mosquitoes were analysed to estimate the human biting rate indoors and outdoors, the circumsporozoite antigen positivity, and the anthropophagic index using ELISA methodology. Polymerase chain reaction was used to estimate the frequency of the knockdown resistance (*kdr*) L1014F and the *ace*-*1* mutations, 2 markers associated respectively with pyrethroids and carbamate/organophosphate insecticide resistance.

**Results:**

A higher blood feeding rate was observed in *An. gambiae* compared to *An. coluzzii* as well as, a non-pronounced outdoor biting behavior in both species. The latter showed similar anthropophagic and sporozoite rates. However the analysis indicates a seasonal difference in the contribution of each species to malaria transmission associated with shifts in resting behaviour. *Anopheles coluzzii* females accounted for most of the detected infections: 86% in Alibori and 79% in Donga, during the dry season *versus* 14.4% and 21.2%, respectively for *An. gambiae* during the same period. This relationship was reversed in Donga during the rainy season (66% for *An. gambiae* against 34% for *An. coluzzii*). Results also indicated lower frequencies of *kdr* L1014F and *ace*-*1* in *An. coluzzii versus An. gambiae.*

**Conclusion:**

Despite similarity in some parameters related to malaria transmission in both surveyed species, *An. coluzzii* is potentially a more important malaria vector because of high density in the region. It is also characterized by lower frequencies of the *ace*-*1* mutation than is *An. gambiae*. The ongoing use of pirimiphos methyl (organophosphate) for IRS should continue to show a good impact in Alibori and Donga because of the very low level of the *ace*-*1* mutation in both species.

## Background

*Anopheles gambiae* sensu lato (s.l.) is a complex of 8 species, which contribute differently to malaria transmission. Within the complex, *An. gambiae* sensu stricto (s.s.), the nominotypical member, is comprised of 2 species based on molecular evidence: *Anopheles coluzzii* (the *An. gambiae* molecular ‘M form’) and *An. gambiae* (the ‘S form’) [1].

Heterogeneity in the vector capacity of each species is due to a highly diverse bio-ecology: feeding on humans or cattle, resting indoors or outdoors. According to Akogbéto [2] and Awolola et al. [3], what makes *An. gambiae* an efficient vector is that it is highly anthropomorphic and endophilic, thereby frequently coming into contact with humans. In addition to its possibility of adaptation to various environments due to its genetic variants [4, 5], *An. gambiae* s.s. is a very efficient vector of malaria, especially in tropical Africa. Nonetheless, other sibling species of *An. gambiae* complex play a role, albeit a lesser role, in malaria transmission, for example, *Anopheles melas* in West Africa [6–8]. Studies on bio-ecology of *An. gambiae* s.l. have shown statistical differences of sporozoite index between different populations sibling species of the complex [9, 10]). Moreover, the main factor of vector competence of a mosquito to a parasite is its ability to offer a suitable physicochemical environment for the development of the parasite during the sporogonic cycle.

The objective of this study was to assess the blood feeding behaviour and the contribution in malaria transmission of each of the 2 sibling species (*An. coluzzii* and *An. gambiae*) present and living in sympatry in 2 regions of Benin, targeted for indoor residual spraying (IRS) on malaria transmission prior to the implementation of IRS. Given that the density of each of the 2 sibling species varies from one season to another, the more abundant species during the 3–6 months period following spraying when IRS residual efficacy operates, should be more influenced by this intervention.

The study is also evaluating, before spraying, the frequency of mutations associated with organophosphates resistance in *An. coluzzii* and *An. gambiae,* two sibling vector species living in sympatry in the study area and which were likely exposed to the same selection pressure for resistance. Indeed, if low frequencies of resistance mechanisms are observed in a given species, this may be sign that IRS with an organophosphate product could have a positive impact on the control of this species as previously observed by Aikpon et al. [11].

Detailed information on bio-ecology of vectors and their role in disease transmission are important for implementation of good vector control strategies or for their evaluation. In the study area (Alibori and Donga region, in the northern Benin) targeted for IRS campaign, where the 2 species are sympatric, the recorded data will be used as the comparison basis of the impact of IRS on each of the 2 *Anopheles* populations.

### Study area

**Fig. 1:**
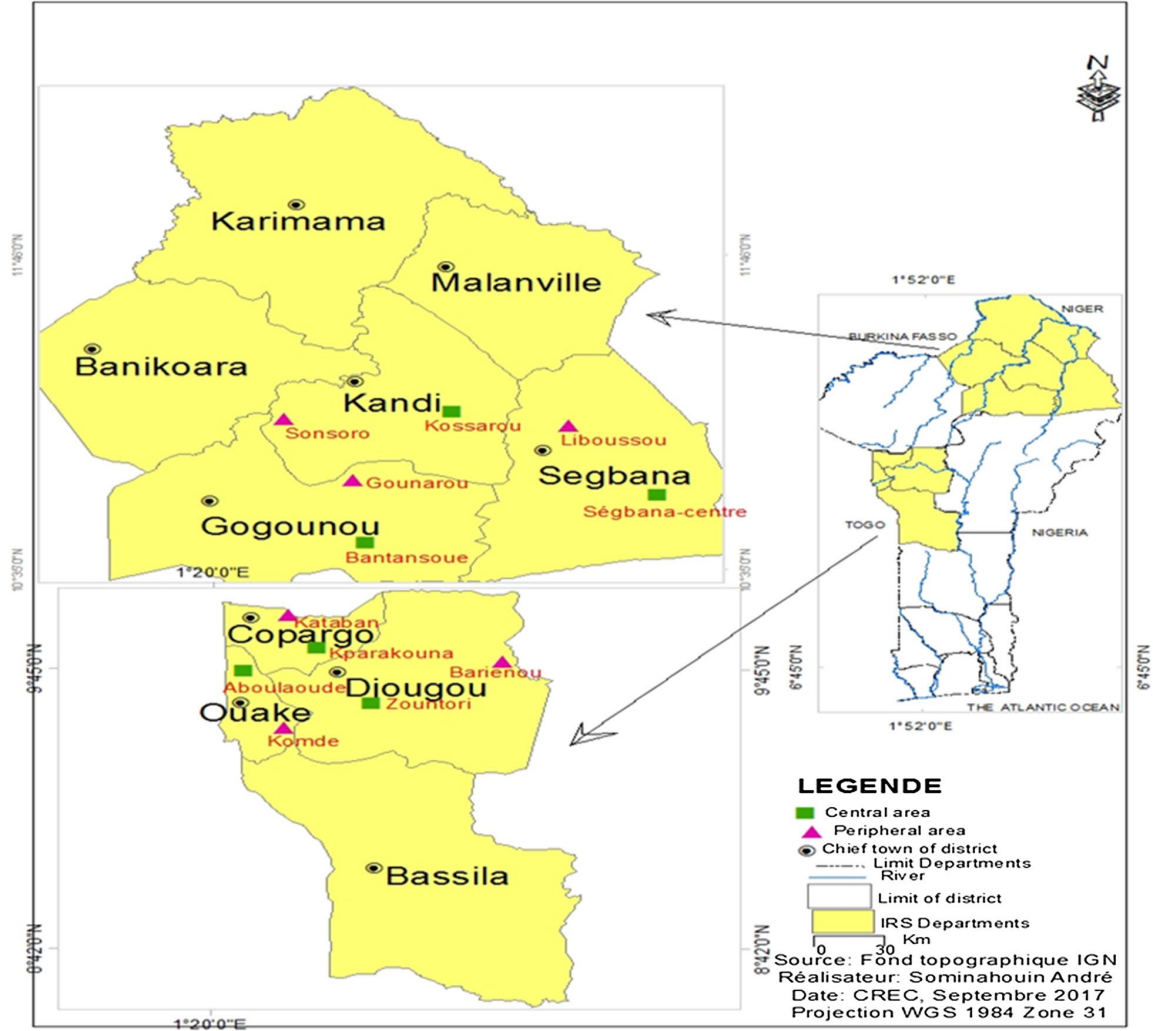
Map of Benin showing the Alibori and Donga regions and the sites of mosquito collections

The study was conducted in 6 districts of the northern Benin: Kandi, Gogounou and Segbana in Alibori region, and Djougou, Copargo and Ouake in Donga region (Fig. 1). The 2 regions are characterized by one dry season (December to May) and one rainy season (June to November). The annual mean rainfall is 1300 mm and the mean monthly temperature varies between 23 and 40 °C. The Alibori region has more rivers than the Donga region. In Alibori, the soil is sandy type while in Donga, it is clayey type. In both regions the major economic activity is agriculture, including the production of cotton, maize and millet, where various classes of pesticides are used for pest control.

### Mosquito sampling and laboratory analysis

HLC carried out indoors and outdoors from 21.00 to 06.00 h and PSC performed inside houses from 06.00 to 09.00 were used to sample vector populations during the rainy (June–October 2016) and dry (November–May 2016) seasons in the 6 districts targeted for IRS implementation.

### Human landing catches (HLC) and mosquito analysis

In each district, mosquito sampling was performed in 2 villages: one village in the central part of the district and one village at the periphery. In each village, mosquitoes were collected by adult volunteers in 2 houses for 2 consecutive nights, every month for 5 months per season, with one adult volunteer placed indoors and another placed outdoors. All *Anopheles* mosquitoes caught during the night were identified to species using the method described by Gilles and de Meillon [12]. To assess the infection status of each species, the head-thoraxes were tested using enzyme-linked immunosorbent analysis (ELISA) according to Wirtz et al. [13] to estimate circumsporozoite (CS) antigen of *Plasmodium falciparum*, the major malaria parasite occurring in the study area. Abdomens from females of the vector species were used for PCR analyses, to identify the sibling species of *An. gambiae* s.s. (*An. gambiae, An. coluzzii*). The indoor and outdoor human biting rate (HBR: also named *ma*) of each species was calculated to estimate the feeding behaviour of the 2 species.

In the study, malaria transmission in both the Alibori and Donga regions is expressed in terms of entomological inoculation rate (EIR = *ma* × *s*) due to both of the 2 species. The contribution of each species is the EIR calculated for the species divided by those of the 2 species × 100.

### Indoor pyrethrum spray catches and mosquito analysis

In each village, 20 houses were selected for mosquito collection using indoor pyrethrum spray catches (PSC). Selected houses were sprayed with pyrethrum (mixed with water) and a white canvas was placed on the floor to collect the mosquitoes that were killed. After 10 min, all of the fallen mosquitoes were collected from the floor and placed in petri dishes. Fed, unfed, gravid, and half-gravid females of *An. gambiae* were counted to estimate the endophagy behaviour, defined as the proportion of each species resting in houses after blood feeding. Fed mosquitoes were also used to estimate the anthropophagic index in terms of the human blood meal rate using ELISA-blood-meal method. The anthropophagic index represents the proportion of blood meals derived from humans by mosquito vectors used to estimate human biting habit.

### PCR detection of *kdr* L1014F and *ace*-*1* mutations

The abdomens of *An. coluzzii* and *An. gambiae* females were used to identify the presence of L1014F (*kdr*) and G119S (*ace*-*1*) mutations. This was performed using PCR–RFLP [14] to estimate the frequency of *kdr* L1014F mutation in the sodium channel, which is associated with resistance to pyrethroid insecticides, and that of the *ace*-*1* mutation, which is associated with carbamate and organophosphate insecticide resistance.

### Statistical analysis

The human biting index (*ma*) (number of *Anopheles* per person and per night), room density (number of *Anopheles* collected in a room) and the circumsporozoite protein (CSP) positive rate were calculated for each species. The human blood-feeding rate was calculated using mosquitoes collected by PSC by dividing the number of fed and half-fed mosquitoes collected by the number of the total of mosquitoes collected. The CSP positive rate (% CS+) was calculated as the proportion of mosquitoes found to be positive for CSP. The EIR was defined as *Anopheles* density by the CSP and estimated as the number of infectious bites per human per night or per month. A Chi square test with the MINITAB statistical software (Version 12.2) was used to compare the proportions. The genotypic differentiation of *kdr* and *ace*-*1* loci was tested using the Fischer exact test implemented in GenePop software [15], and the Fisher test was used to compare these frequencies. An analysis of variance (ANOVA) was performed to compare the entomological estimates (*ma*, EIR, CSP) among the 2 species.

### Ethical consideration

Permission was sought from households to perform collections in their rooms. In addition, community consent had been obtained beforehand in all the villages. The volunteer mosquito collectors gave their consent before participating in the study. They were also subjected to regular medical check-ups with preventive malaria treatment. They were all vaccinated against yellow fever. This study received the approval of the Ethical Institutional Committee of the Centre for Entomological Research of Cotonou (CREC), Ministry of Health.

## Results

### Distribution of *Anopheles gambiae* complex in the two regions

**Fig. 2:**
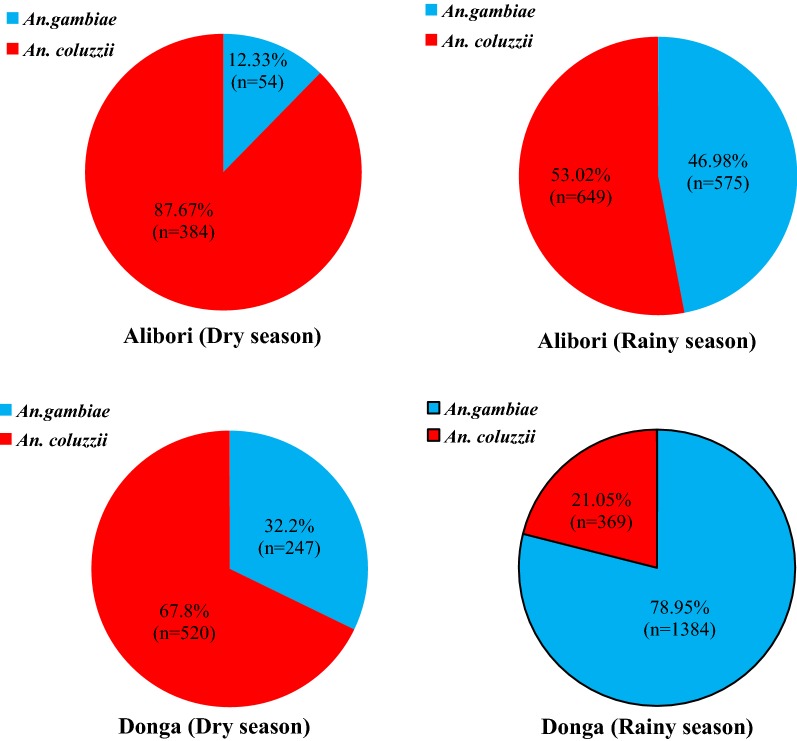
Seasonal distribution of *Anopheles coluzzii* and *Anopheles gambiae* in Alibori and Donga, two regions of northern Benin

All samples of *An. gambiae* s.l. collected by HLC and PSC were used for species identification. In total, 4182 *An. gambiae* s.l. were analysed by PCR for species identification. Two species of the *An. gambiae* s.s. were identified: *An. coluzzii* and *An. gambiae* (Fig. 2). After 1 year of mosquito collection throughout both dry and rainy seasons, *An. gambiae* represented 54.0% (n = 2260) of the overall *An. gambiae* s.s. population compared to 46.0% (n = 1922) for *An. coluzzii*. However, the predominant species differed from one region to another. In Alibori, *An. coluzzii* represented 62.2% (n = 1033) of the overall *An. gambiae* s.s. population compared to 37.8% (n = 625) for *An. gambiae*. In Donga, *An. gambiae* was the most abundant at 64.7% (n = 1631).

Both of the sibling species were present throughout the dry and rainy seasons in both regions (Fig. 2). During the dry season (January to June), *An. coluzzii* predominated in both the Alibori and Donga regions. However, during the rainy season, there were regional differences in the predominant species: during the rainy season in Donga, there was a high frequency (79.0%) of *An. gambiae* whereas during the rainy season in Alibori there was a relatively high frequency (53.0%) of *An. coluzzii* (Fig. 2).

### Comparison of (CS) antigen index for *Plasmodium falciparum* in *Anopheles gambiae* and *Anopheles coluzzii* during the dry and rainy seasons

More than 4000 head-thoraxes of *An. gambiae* and *An. coluzzii* were tested using ELISA [13] for the presence of CS of *P. falciparum.* Various samples of *An. gambiae* (from HLC and PSC collection) were analysed in order to obtain enough mosquitoes (4182 head-thoraxes) for comparison.

During the dry season, rates of *P. falciparum* CS + were similar for *An. gambiae* (9.3%: 5 out of 54 thoraxes were CS+) and *An. coluzzii (*5.5%: 21 out of 384) (p = 0.426) in Alibori. Results were statistically different in Donga during the dry season with a higher rate of *P. falciparum* CS + for *An. coluzzii* (9.6%: 50 out of 520) compared to *An. gambiae* (4.9%: 12 out of 247) (p = 0.002) (Table 1).

**Table 1:** Percentage of *P. falciparum* circumsporozoite antigen in *An. coluzzii* and *An. gambiae* during the dry and the rainy seasons

Regions	Species		Dry season (May–Jun–Jan–Feb)	p-value	Rainy season (Jul–Aug–Oct)	p-value	Total	p-value
Alibori	*An. gambiae*	Thorax	54	0.426	575	0.096	629	0.55
		Thorax+	5		42		47	
		Is (%)	9.26		7.30		7.47	
		IC-95% [IS %]	[3.07–20.30]		[5.31–9.75]		[5.54–9.81]	
	*An. coluzzii*	Thorax	384		649		1033	
		Thorax+	21		66		87	
		Is (%)	5.47		10.17		8.42	
		IC-95% [IS %]	[3.41–8.24]		[7.95–12.76]		[6.80–10.28]	
Donga	*An. gambiae*	Thorax	247	0.002	1384	1	1631	0.59
		Thorax+	12		132		144	
		Is (%)	4.86		9.54		8.83	
		IC-95% [IS %]	[2.53–8.33]		[8.04–11.21]		[7.49–10.31]	
	*An. coluzzii*	Thorax	520		369		889	
		Thorax+	50		35		85	
		Is (%)	9.62		9.49		9.56	
		IC-95% [IS %]	[7.22–12.48]		[6.69–12.94]		[7.7–11.69]	
Alibori-Donga	*An. gambiae*	Thorax	301	0.252	1959	0.389	2260	0.607
		Thorax+	17		174		191	
		Is (%)	5.65		8.88		8.45	
		IC-95% [IS %]	[3.32–8.89]		[7.65–10.23]		[7.33–9.67]	
	*An. coluzzii*	Thorax	904		1018		1922	
		Thorax+	71		101		172	
		Is (%)	7.85		9.92		8.94	
		IC-95% [IS %]	[6.18–9.80]		[8.15–11.92]		[7.71–10.31]	

p = p-value of comparison of *CircumSporozoite* (CS) *antigen index for Plasmodium falciparum* of *An. gambiae* and *An. coluzzii*

*Jun* June, *Jul* July, *Aug* August, *Oct* October, *Jan* January, *Feb* February

During the rainy season, rates of *P. falciparum* CS + were similar for both species in Alibori (*An. gambiae*: 7.3%: 42 out of 575) thoraxes were CS + ; *An. coluzzi*: 10.2%, 66 out of 649) (p = 0.096) and in Donga (*An. gambiae*: 9.5%, 132 out of 1384; *An. coluzzi:* 9.5%, 35 out of 369) (p = 1) (Table 1).

No differences in CS + between species were observed in either region when data were cumulated over both seasons: 7.5%: 47 out of 629 thoraxes were CS + for *An. gambiae* and 8.4%: 87 out of 1033 for *An. coluzzii* in Alibori (p = 0.55). In Donga, CS + for *An. gambiae* and *An. coluzzii* were 8.8% (144 out of 1631) and 9.6% (85 out of 889), respectively (p = 0.59) (Table 1).

In total, 191 (8.5%) of 2260 thoraces of *An. gambiae* analysed in the two regions by ELISA-CSP were positive for *P. falciparum* CS antigen (Is = 8.5%). For *An. coluzzii*, 172 (8.9%) of 1922 thoraces were positive for *P. falciparum* CS antigen (p = 0.60) (Table 1).

### Contribution of *Anopheles gambiae* and *Anopheles coluzzii* to malaria transmission expressed in terms of the EIR

The intensity of the malaria transmission due to each species is expressed in terms of EIR, which is equal to *ma* times the sporozoite rate. As the *ma* parameter depends on the season, the EIR due to each species was assessed for the dry season when *ma* is expected to be low and for the rainy season when *ma* is expected to be high.

**Fig. 3:**
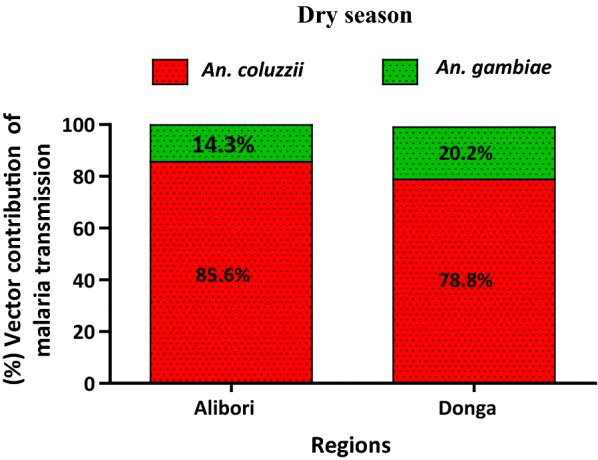
Contribution of *An. coluzzii* and *An. gambiae* expressed in the proportion of Entomological Inoculation Rate due to each species in Alibori and Donga during the dry season

In both the Alibori and Donga regions, *An. coluzzii* accounts for most malaria transmission during the dry season. In Alibori, *An. coluzzii* accounts for 85.6% of malaria transmission during the dry season (EIR = 1.61 infected bite per man per month) compared to 14.3% (EIR = 0.27/month). In Donga, *An. coluzzii* and *An. gambiae* account for 78.8% (EIR = 3.20/month) and 20.2% (EIR = 0.81/month) of malaria transmission in the dry season, respectively (Fig. 3).

**Fig. 4:**
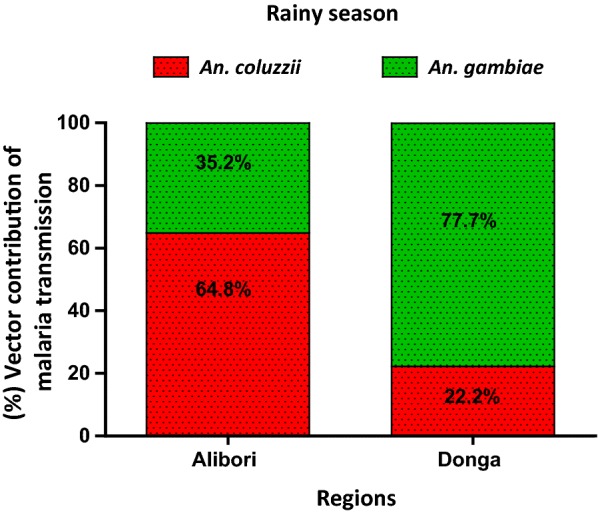
Contribution of *An. coluzzii* and *An. gambiae* expressed in the proportion of Entomological Inoculation Rate due to each species in Alibori and Donga during the rainy season

**Fig. 5:**
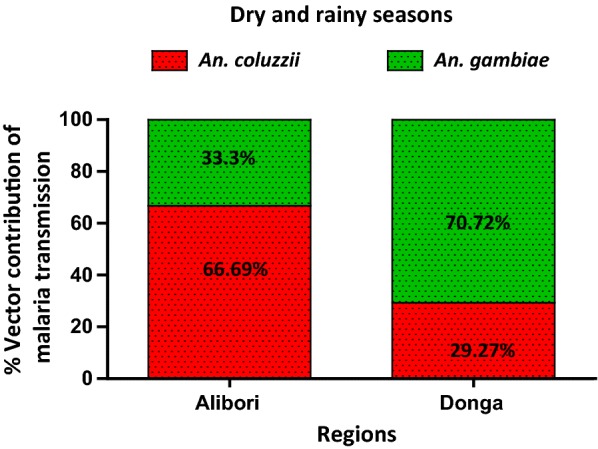
Contribution of *An. coluzzii* and *An. gambiae* expressed in the proportion of Entomological Inoculation Rate due to each species in Alibori and Donga during the two seasons

During the rainy season, *An. coluzzii* continues to account for most malaria transmission in the Alibori region as occurred during the dry season: *An. coluzzii* accounts for 64.8% (14.37 infected bites) of malaria transmission compared to 35.2% (7.81 infected bites) for *An. gambiae* (Table 2). However, in the Donga region, inverted results were observed showing a higher EIR due to *An. gambiae* (77.7%: EIR = 26.25 infected bites/month) as compared to *An. coluzzii* (Fig. 4). When cumulated data, which were registered during the 7 months of the study, the observed EIR trend during the dry season was confirmed due to the high *ma* of *An. coluzzii* during this period: the EIR of *An. coluzzii* was 2 times higher than *An. gambiae* in Alibori (p = 0.0043) and 2 times lower in Donga (p = 0.000084) (Fig. 5).

**Table 2:** Malaria transmission expressed in terms of Entomological Inoculation Rate (EIR) in *An. coluzzii* and *An. gambiae* in Alibori and Donga

Regions	Species		Dry season (May–Jun–Jan–Feb)	p-value	Rainy season (Jul–Aug–Oct)	p-value	Total	P-value
Alibori	*An. gambiae*	Thorax	12	0.125	362	*0.016*	374	*0.0043*
		Thorax+	1		25		26	
		Is (%)	8.33		6.91		6.95	
		ma/month	3.21		113.12		53.94	
		EIR/month	0.27		7.81		3.74	
	*An. coluzzii*	Thorax	135		446		581	
		Thorax+	6		46		52	
		Is (%)	4.44		10.31		8.95	
		ma/month	36.16		139.37		83.79	
		EIR/month	1.61		14.37		7.49	
Donga	*An. gambiae*	Thorax	147	*0.03*	846	*0.000054*	993	*0.000084*
		Thorax+	3		84		87	
		Is (%)	2.04		9.93		8.76	
		ma/month	1.31		264.37		143.22	
		EIR/month	0.810		26.25		12.54	
	*An. coluzzii*	Thorax	214		257		471	
		Thorax+	12		24		36	
		Is (%)	5.607		9.339		7.643	
		ma/month	57.32		80.31		67.93	
		EIR/month	3.20		7.50		5.19	

Italic values indicate significance of p value (p < 0.05)

p = p-value of comparison of *CircumSporozoite* (CS) antigen index for *Plasmodium falciparum* of *An. gambiae* and *An. coluzzii*

*Jun* June, *Jul* July, *Aug* August, *Oct* October, *Jan* January, *Feb* February

### Indoor and outdoor biting behaviour of *Anopheles gambiae* and *Anopheles coluzzii*

More than 2400 *An. gambiae* collected by HLC and analysed by PCR showed that, the biting behaviour of *An. gambiae* and *An. coluzzii* was variable according to the location (indoors or outdoors). Out of 1302 *An. gambiae* collected by HLC in Alibori and Donga, 57.7% (752/1302) were collected indoors compared to 42.3% (550/1302) outdoors (p < 0.001) (Table 3). As for *An. coluzzii,* a similar biting behaviour was observed indoors and outdoors with respectively: 51.6% (575/1114) and 48.4% (539/1114) of specimens (p = 0.13), after cumulating data of both regions (Table 3). During the dry season, the biting behaviour of *An. coluzzii* was higher indoors with 56.4% (216/383) of specimens as compared to outdoors (43.6%) (p = 0.0004). In the same season, the biting behavior was similar indoors and outdoors (p = 0.25) for *An. gambiae* (Table 3).

**Table 3:** Number of *An. gambiae* and *An. coluzzii* collected indoors and outdoors by HLC

	Seasons	Species	Indoors	Outdoors	p-value
Alibori	Dry season	*An. gambiae* (N)	7	7	–
		*An. coluzzii* (N)	59	74	–
	Rainy season	*An. gambiae* (N)	212	164	–
		*An. coluzzii* (N)	256	176	–
Donga	Dry season	*An. gambiae* (N)	60	51	–
		*An. coluzzii* (N)	157	93	–
	Rainy season	*An. gambiae* (N)	472	329	–
		*An. coluzzii* (N)	103	196	–
Both regions	Dry season	*An. gambiae* [% (N/T)]	53.6% (67/125)	46.4% (58/125)	0.25
		*An. coluzzii* [% (N/T)]	56.4% (216/383)	43.60% (167/383)	*0.0004*
	Rainy season	*An. gambiae* [% (N/T)]	58.11% (684/1177)	41.88% (493/1177)	*< 0.001*
		*An. coluzzii* [% (N/T)]	49.11% (359/731)	50.88% (372/731)	0.5
Both regions	Both seasons	*An. gambiae* [% (N/T)]	57.7% (751/1302)	42.3% (551/1302)	*< 0.001*
		*An. coluzzii* [% (N/T)]	51.6% (575/1114)	48.4% (539/1114)	0.13

Italic values indicate significance of p value (p<0.05)

*N* number of species collected in one location (indoors or outdoors), *T* total number of species collected in both locations (indoors + outdoors)

During the rainy season, the trend was reversed with a similar biting behaviour indoors and outdoors (p = 0.5) for *An. coluzzii* and, a higher indoor biting behaviour for *An. gambiae* (Table 3).

### Blood feeding rate and anthropophagic index

With PSC performed in bedrooms, a significant higher blood feeding rate [96.68% (846 of fed and half-gravid females out of 875 total females)] was observed in *An. gambiae* as compared to *An. coluzzii* [90.20% (792/878)] (p < 0.0001) (Table 4). A high and similar anthropophagic index (0.97) was observed for both species with 34/35 *An. gambiae* specimens and 33/34 *An. coluzzii* having fed on humans (p = 0.98).

**Table 4:** Blood feeding rate of *An. gambiae* and *An. coluzzii* collected by PSC method in Alibori and Donga

Regions	*An. gambiae*						*An. coluzzii*						p-value
	Total collected	Unfed	Fed	Gravid	Half-gravid	Blood feeding rate (%)	Total collected	Unfed	Fed	Gravid	Half-gravid	Blood feeding rate (%)	
Alibori	235	5	216	3	11	96.6	460	20	358	35	47	88.04	*0.0003*
Donga	640	13	604	8	15	96.72	418	11	347	20	40	92.58	*0.003*
Both regions	875	18	820	11	26	96.68	878	31	705	55	87	90.20	*<* *0.0001*

Italic values indicate significance of p value (p<0.05)

p-value: p-value of comparison of the feeding rate of *An. gambiae* to that of *An. coluzzii* by region

### Frequency of *kdr* mutation in *Anopheles gambiae* and *Anopheles coluzzii* populations

A total of 557 females of *An. gambiae* and 423 *An. coluzzii* were analysed for L1014F *kdr* mutation and G119S mutation (*ace*-*1* gene) using PCR–RFLP [14]. The frequency of the 2 mutations is higher in *An. gambiae* (0.82 for *kdr* and 0.03 for *ace*-*1*) than in *An. coluzzii* (0.73 for *kdr* and 0.01 for *ace*-*1*) (Table 5).

**Table 5:** Distribution of Knock-down resistance (*Kdr*) and *Ace*-*1R* frequencies in *An. gambiae* and *An. coluzzii* in Alibori and Donga

Regions	Species	Number tested	*Mutation KdrL1014F*					*Mutation Ace 1R*				
			RR	RS	SS	F (Kdr)	p-value	RR	RS	SS	F (Ace 1)	p-value
Alibori	*An. gambiae*	170	123	34	13	0.82	*0.0024*	0	10	160	0.03	0.1381
	*An. coluzzii*	271	143	90	38	0.69		0	7	264	0.01	
Donga	*An. gambiae*	387	272	93	22	0.82	0.153	0	20	367	0.03	0.2981
	*An. coluzzii*	152	102	34	16	0.78		0	4	148	0.01	
Total	*An. gambiae*	557	395	127	35	0.82	*0.0032*	0	30	527	0.03	*0.0483*
	*An. coluzzii*	423	245	124	54	0.73		0	11	412	0.01	

Italic values indicate significance of p value (p < 0.05)

p = p-value of comparison of the frequencies *Kdr* and *Ace*-*1* between *An. gambiae* and *An. coluzzii*

## Discussion

In Africa, vector surveillance is an integral component to the planning, implementation, monitoring, and the evaluation of vector control interventions. To assist with this goal, the current study was initiated in an area of Benin targeted for an IRS campaign to provide baseline data that could be used to help evaluate the impact and efficacy of IRS in the future.

*Anopheles gambiae* complex is the most important malaria vector in sub-Saharan Africa and has 4 species that occur in West Africa: *An. coluzzii*, *An. gambiae*, *An. arabiensis* and *An. melas* [4–8]. In the area investigated by the current study only 2 species maintain malaria transmission: *An. gambiae* and *An. coluzzii*. Both species are present year-round with a strong predominance of *An. coluzzii* in dry season. Despite the 2 study areas having a similar climate, there was a disparity in the distribution of the 2 species during the rainy season with a strong occurrence of *An. coluzzii* in the Alibori region compared with the Donga region. The low proportions of *An. gambiae* in the dry season may be due to temporary breeding sites that dry up, in addition to the presence of permanent and semi-permanent breeding sites more favourable to the development of *An. coluzzii* larvae.

Despite the large number of *An. gambiae s.s*. specimens (more than 4000) collected by HLC and PSC that were analysed by PCR, no *An. arabiensis* was found. Only one specimen of *Anopheles nili* was morphologically identified in Alibori and Donga. The absence of *An. arabiensis* in the study area could be due to the exophilic and zoophilic behaviour of this species and to its gradual disappearance in some environments in West Africa [4, 16], particularly in northern Benin [6]. Indeed, in 1992, Akogbeto [2] reported its presence in sympatry with *An. gambiae* s.s. in northern Benin, but in 2010, Aïkpon et al. [17] reported its disappearance in the same region.

The overall biting rate of *An. gambiae* and *An. coluzzii* was not higher outdoors than indoors in both the rainy and dry seasons, which suggests an important role for ITNs in the effort to prevent malaria. However, despite the presence of ITNs in houses in the study area, *An. gambiae* s.s. maintained a high index of human-vector contact as indicated by high human biting rates, which could be a result of its resistance to the pyrethroid insecticides used to treat bed nets.

The significant higher blood feeding rate observed in *An. gambiae* as compared to *An. coluzzii* indoors (p < 0.0001) would be likely due to a higher ability of *An. gambiae* to bite and feed on humans inside houses. Indeed, the collected HLC data show an overall more pronounced indoor man-vector contact in *An. gambiae* (56.6%: 751/1326) as compared to *An. coluzzii* (43.4%: 575/1326) (p < 0.0001). This result is similar to that obtained in Ghana by Tuno et al. [18] who showed that *An. gambiae* bit people inside dwellings more than did *An. melas*.

The fact that, the majority (≥ 90%) of *An. coluzzii* and *An. gambiae* collected indoors were blood-fed with a high and similar anthropophagic index (0.97), mirrors results from similar studies [17, 19–23]. As a result of both species having similar attractions to humans, *An. gambiae* and *An. coluzzii* had similar sporozoitic indexes at 8.5 and 8.9%, respectively (p = 0.60). This result is reminiscent of that of Carnevale et al. [24] who showed the same infection rate in *An. coluzzii* and *An. gambiae* in Lobito, Angola. Given the similar affinity for humans and the large number of specimens (more than 4000) examined through ELISA-CSP, it is likely that *An. gambiae* and *An. coluzzii* have the same ability to offer a physicochemical environment suitable for the development of the parasite during the sporogonic cycle.

By taking into account the fact that vector capacity of a population of *Anopheles* depends on several factors, in particular, the ability to foster the development of the sporogonic cycle, the contribution of *An. coluzzii* and *An. gambiae* to malaria transmission was expressed in terms of the sporozoitic index (*s*) and the frequency of human-vector contacts (*ma*). Based on the calculated EIRs, *An. coluzzii* is involved in 85.6% of malaria transmission in Alibori and 78.8% of transmission in the Donga during the dry season, compared to only 14.4 and 21.2% for *An. gambiae* in Alibori and Donga, respectively. Further, during the rainy season *An. coluzzii* continues to account for most malaria transmission in Alibori (64.8% compared to 35.2% for *An. gambiae*), but the role is reversed in Donga during the rainy season with a higher contribution toward malaria transmission from *An. gambiae* (65.7% compared to 34.3% for *An. coluzzii*). The variation in abundance of each vector species from one season to another could explain this. Indeed, Alibori and Donga regions are crossed by several rivers and water dams that create throughout the year (rainy and dry seasons), permanent and semi-permanent breeding sites suitable for the development of *An. coluzzii* larvae. In addition, the Donga region is characterized by a clayey type soil, which retains water, allowing then the formation of numerous temporary breeding sites favourable to the emergence of *An. gambiae* as leading vector of malaria transmission in the rainy season. By against, in Alibori, the sandy type soil causes the quick infiltration of water and as a result, fewer temporary larval habitats are formed, which justifies that *An. coluzzii* coming from permanent and semi-permanent breeding sites continues to play the major role in malaria transmission during the rainy season in this region. As observed in Donga from dry to rainy season, the switch of the mosquito species that leads malaria transmission has also been reported in Lobito, Angola from 2005 to 2006 and, the suspected causes were environmental anthropic modifications (installation of water cisterns that disrepair over time and are used as dumps, reduction of some artificial larval habitats types favourable to *An. gambiae*) [24].

There have been numerous reports of resistance to pyrethroid throughout Africa [25–30]. In Benin, the resistance of malaria vectors to pyrethroids observed first in Cotonou spread not only to central and southern regions of the country, but also to the northern parts [31–38]. The lower frequency of *kdr* L1014F mutation in *An. coluzzii* in Alibori could favour the impact of ITNs and result in better control of malaria in Alibori compared to Donga. However, if pirimiphos methyl (organophosphate) is used again for IRS in the study area as it was in previous years, a positive impact could be expected because of the very low level (0.01–0.03) of the *ace*-*1* mutation in both species.

## Conclusion

*Anopheles coluzzii* and *An. gambiae* have been observed to have the same anthropophagic and sporozoite rates in the 2 regions. However, *An. coluzzii* is highly involved in malaria transmission in Alibori and Donga during the dry season as compared to *An. gambiae*. During the rainy season *An. coluzzii* was still playing the leading role in Alibori whereas, in Donga, this role is reversed with a stronger participation of *An. gambiae*. Overall, the blood feeding rate was higher in *An. gambiae* than in *An. coluzzii.* Moreover, data collected reveal *An. coluzzii* is characterized by lower frequencies of the *ace*-*1* mutation than is *An. gambiae*. But, a good impact on malaria control in Alibori and Donga is expected if pirimiphos methyl (organophosphate) is used again for IRS in Benin as in previous years, because of the very low level of the *ace*-*1* mutation in both species.
